# NADPH oxidase expression and production of superoxide by human corneal stromal cells

**Published:** 2009-12-03

**Authors:** William J O’Brien, Tom Heimann, Farhan Rizvi

**Affiliations:** 1 Department of Ophthalmology, Medical College of Wisconsin, Milwaukee, WI; 2 Department of Microbiology/Molecular Genetics, Medical College of Wisconsin, Milwaukee, WI

## Abstract

**Purpose:**

Superoxide (O_2_^.-^) may function as a second messenger or regulator of signal transduction when produced at low concentrations in the proper locations within cells. The purpose of these studies was to determine whether human corneal stromal (HCS) fibroblasts are capable of producing O_2_^.-^ via nicotinamide adenine dinucleotide phosphate (NADPH) oxidases, a family of protein complexes believed to be responsible for the localized and limited production of O_2_^.-^ with regulatory activity.

**Methods:**

HCS cells, grown as primary and low-passage cultures of fibroblasts, were used as the sources of RNA for reverse transcriptase PCR, with primers specific for mRNAs encoding the proteins that comprise NADPH oxidases. Small interfering (si)RNAs were used to knockdown specific *NOX* mRNAs. Proteins composing the NADPH oxidase complexes were identified using western blots. The production of O_2_^.-^ by whole cells and cell-free preparations was assessed by measurement of NADPH-dependent superoxide dismutase-inhibitable cytochrome *c* reduction.

**Results:**

Whole cells and cell-free extracts of corneal stromal fibroblasts produced O_2_^.-^ in an NADPH-dependent manner. These fibroblasts constitutively produced mRNAs encoding eight proteins known to comprise NADPH oxidase complexes. mRNAs encoding *NOX1*, *NOX4*, *NOX5*, *p22 phox*, *p47 phox*, *p67 phox*, and *p40 phox* as well as *Rac* were expressed. Treatment of HCS fibroblasts with siRNA pools specific for each of these three NOXs significantly reduced the steady state levels of the respective mRNAs. Western blots confirmed the existence of all the proteins required for O_2_^.-^ production. Rac 1, a regulator of the activity of some forms of NADPH complexes was present in membranous cell fractions containing the oxidase proteins.

**Conclusions:**

HCS fibroblasts produced O_2_^.-^ in a NADPH-dependent manner via at least three isoforms of NADPH oxidase. These cells expressed *NOX1*, *NOX4*, *NOX5*, *p22 phox*, *p47 phox*, *p67 phox*, and *p40 phox* as well as *Rac*. SiRNAs directed against each of the three putative isoforms of NOX significantly reduced the steady state levels of the appropriate *NOX* mRNA pools, thus confirming the existence of the three isoforms. The O_2_^.-^ produced by the NADPH oxidases in HCS fibroblasts is a potential contributor to signal transduction pathways and a regulator of gene expression as well as a potential participant in processes that occur during inflammation.

## Introduction

Superoxide anion (O_2_^.-^) is one of a group of molecules referred to as reactive oxygen species (ROS). The production of low amounts of O_2_^.-^ by nicotinamide adenine dinucleotide phosphate (NADPH) oxidase in a highly regulated and localized manner can play critical roles in cell survival and death [1-5]. O_2_^.-^ may be produced directly by enzyme activity or by uncoupled enzymes (i.e., indirectly by enzymatic reactions where the reduction of molecular oxygen is no longer directly linked to product formation). Superoxide can be produced in cells by several enzyme systems, including xanthine oxidase, uncoupled nitric oxide synthase, complexes I or III of the mitochondrial electron transport system, uncoupled NADPH cytochrome P450 reductase, or NADPH oxidases [6-9].

The NADPH oxidase family currently contains seven members composed of multiple proteins that may exist in several isoforms. The NADPH oxidase of neutrophils is the classic membrane-bound complex that is assembled upon exposure of the cells to activating stimuli, such as bacterial lipopolysacchride. The complex in neutrophils contains the NOX2 protein and is capable of the production of large amounts of O_2_^.-^ [10]. This oxidase consists of NOX2 complexed with p22 phox, p47 phox , p67 phox, p40 phox, and Rac2. The complex is assembled at the cytoplasmic membrane when the cell receives the proper stimulus. NADPH oxidase complexes are also responsible for the production of O_2_^.-^ in several other cell types, including cardiac myocytes, dermal epithelial cells, and kidney epithelial cells [4,11,12]. Some cells contain homologues of the classic NADPH oxidase proteins. The proteins of these homologous complexes, as they exist in nonmyeloid cells, may contain any of several isoforms of NOX, p47 phox, or p67 phox. Cells containing complexes that are homologues of the NOX2 oxidase produce relatively small amounts of O_2_^.-^ [13]. Cells other than leukocytes and macrophages may express NOX proteins (NOX1-5) as well as isoforms of p47 phox, p40 phox, and p67 phox and Rac1 rather than Rac2 [14-16]. Several functions have been proposed for the O_2_^.-^ produced by these NADPH complexes, including roles in apoptosis, senescence, cell proliferation, oxygen sensing, signal transduction, and regulation of hormone synthesis [1,4,17].

In the human cornea multiple potential sources of O_2_^.-^ exist not only during inflammation but also in healthy tissue. Enzymes, such as xanthine oxidase, uncoupled NADPH cytochrome P450 reductase, nitric oxide synthase 2 (NOS2), and complexes I and III of the mitochondrial electron transport chain are all capable of O_2_^-^ production in corneal cells [8,18-20]. Cells derived from eyes, including rabbit corneal epithelial and stromal cells as well as human lens epithelial cells and retinal vascular endothelial cells, contain modified forms of NADPH oxidase complexes [20-23]. The expression of NADPH oxidases has not been previously demonstrated in human corneal stromal (HCS) cells, although O_2_^.-^ has been hypothesized to contribute to keratoconus [24]. The results of the studies reported here document that HCS cells are capable of producing O_2_^.-^ by the oxidation of NADPH via NADPH oxidases. The results demonstrate that the oxidases exist as an NOX5 oxidase as well as NOX1 and NOX4 complexed with p22 phox, p47 phox, p40 phox, p67 phox, or their homologues and Rac.

## Methods

### Cell culture

HCS cells were isolated, grown to confluence, and subcultured, using modifications to published procedures [25]. Corneal stromal cells were isolated from excised corneas after removal of the epithelium and endothelium by scraping. The stroma was digested for 16 h at 37 °C with 150 units/ml collagenase (*Clostridium* *histolyticium*, Invitrogen, Carlsbad, CA) in Hank’s balanced salts solution (HBSS) containing penicillin G (100 units/ml) and streptomycin sulfate (100 μg/ml; both from Sigma Aldrich, St. Louis, MO). The cells were recovered by centrifugation (800× g), suspended in growth medium, and grown at 34 °C in Dulbecco’s Modified Eagle Medium (DMEM) containing 4.5 g/l glucose (Invitrogen), 5% heat-inactivated defined fetal bovine serum (FBS; Hyclone/Thermo Scientific, Waltham, MA), 0.1% Mito+serum extender (BD Biosciences, San Jose, CA), and 10 μg/ml ciprofloxacin (Sigma Aldrich). Cells were passaged at a 1:3 split ratio using 0.05% trypsin and 0.53 mM EDTA. Cells were used in the first four passages.

### Reverse transcriptase PCR and real-time PCR

Total RNA was prepared using Trizol (Invitrogen) followed by DNA digestion with DNase I (Invitrogen). Total RNA concentration was determined by measuring the optical density at 260 nm. cDNA synthesis was performed using Superscript III (Invitrogen) and 1-2 μg of RNA. cDNA as amplified using AmpliTaq Gold (Applied Biosystems, Foster City, CA) and sequence-specific primers for standard reverse transcriptase (RT)-PCR (Table 1) and real-time PCR (Table 2). Approximately 4-10% of the RT reaction mixture was used in each PCR reaction containing 1-3 mM MgCl_2_, 200 µM each deoxynucleotide triphosphates (dNTPs), 0.2 to 0.4 µM primers, and 0.05 units/μl units AmpliTaq Gold in reaction buffer (10 mM Tris-HCl, pH 8.3, and 50 mM KCl). The PCR was run at 95 °C for 10 min then 40 cycles of 95 °C for 1 min, 54 to 62 °C for 1 min, and 72 °C for 1 min. Control reactions were run in the absence of the RT in order to detect the presence of amplifiable DNA that might be contaminating the RNA preparation. The reaction products were detected on ethidium bromide-stained NuSieve (2%) agarose gels (Cambrex Bioscience, East Rutherford, NJ).

**Table 1:** Primers for the detection of NADPH oxidase and other cDNAs in human corneal stromal fibroblasts.

**Primer name**	**Sequence 5’→3’**	**Product (bp)**	**Reference**
HuNOX1-S1	GCCTGTGCCCGAGCGTCTGC	522	AJ438989
HuNOX1-AS2	ACCAATGCCGTGAATCCCTAAGC		
HuNOX2-S1	GGAGTTTCAAGATGCGTGGAAACTA	549	[14]
HuNOX2-AS2	GCCAGACTCAGAGTTGGAGATGCT		
HuNOX3-S1-2	CCATGGGACGGGTCGGATTGT	376	NM_015718
HuNOX3-AS2-2	GGGGGCAGAGGTAAGGGTGAAGG		
HuNOX4-S1	GTCATAAGTCATCCCTCAGA	797	[45]
HuNOX4-AS2	TCAGCTGAAAGACTCTTTAT		
NOX5-S1	ATCAAGCGGCCCCCTTTTTTCAC	238	[14]
NOX5-AS2	CTCATTGTCACACTCCTCGACAGC		
P22PHOX-S1	GTTTGTGTGCCTGCTGGAGT	316	[46]
P22PHOX-AS2	TGGGCGGCTGCTTGATGGT		
P47PHOXR-S1	CTCCCGCTGTCCACACCTGCTGAA	349	AF324409
P47PHOXR-AS2	GGGCTCTGGGTCCTCTGGCTCGTC		
P67PHOX-S1	TACTTCCAACGAGGGATGCTC	714	[47]
P67PHOX-AS2	AGCTTTCCTCCTGGGCT		
P67PHOXR-S1	CACGCCCGGATCTGCTTCAAC	363	AF323789
P67PHOXR-AS2	GTGGCGGGGCTCGACTTCAT		
P40PHOXR-S1	TCATCTACCGCCGCTACCGCCAGTTC	473	AF323790
P40PHOXR-AS2	AGTGCCCTCCAGCCAGTCCTTGTTGA		
P40PHOXR2-S1	CCGGAGGAAGATGACCCCACCAACTG	249	AF323790
P40PHOXR2-AS2	GCCTCGCCTGCCTCCACCAT		
MuRAC1-S1	CCTACCCGCAGACAGTTGGAGACAC	395	XM_132485
MuRAC1-AS2	CTTGACAGGAGGGGGACAGAGAACC		
MuRAC2-S1	GCCCCAGCACCCCCATCATCC	252	NM_009008
MuRAC2-AS2	AGGGGCGCTTCTGCTGTCGTGTG		
MuRAC3-S1	GCTTCGGCCACTCTCCTATCCTCA	202	AB040819
MuRAC3-AS2	CTTCTTGTCCCGCAGCCGTTCA		
MamGAPDH-S1	CCATGGAGAAGGCTGGGG	196	[48]
MamGAPDH-AS2	CAAAGTTGTCATGGATGACC		

**Table 2:** Real time PCR primers.

**Primer name**	**Sequence 5'→3'**	**Product (bp)**	**Reference**
GAPDH-S1	CTCCTGCTCCTCTGTTCG	100	NM_002046
GAPDH-AS-1	TAGCTCCGACCTTCACCTTC		
HuNOX1-S1	CACAAGAAAAATCCTTGGGTCAA	106	NM_013955
HuNOX1-AS1	GACAGCAGATTGCGACACACA		
HuNOX4-S1	GCAGGAGAACCAGGAGATTG	125	NM_016931
HuNOX4-AS1	CACTGAGAAGTTGAGGGCATT		
HuNOX5-S1	GCAGGAGAAGATGGGGAGAT	100	NM_024505
HuNOX5-AS1	CGGAGTCAAATAGGGCAAAG		
p22phox-S1	CGCTGGCGTCCGCCTGATCCTCA	128	[26]
p22phox-AS1	ACGCACAGCCGCCAGTAGGTAGAT		

Real-time quantitative amplifications were performed in an ABI 7500 (Applied Biosystems), using SYBR Green PCR Master Mix (Applied Biosystems) and 50-75 ng cDNA at 50 °C for 2 min, 95 °C for 10 min, 40 cycles of 95 °C for 15 s, annealing for 1 min at 60 °C, then extension at 72 °C for 45 s. Primer concentrations for real-time PCR were 10 pM/20 μl. Melt curve analysis was performed by an additional dissociation step of 1 cycle at 95 °C for 15 s and ramping data collection at 60 °C for 1 min and 95 °C for 15 s. Data were normalized against the glyceraldehyde-3-phosphate dehydrogenase (*GAPDH*) signal. Primers were either designed by us or as published by others (Table2) [26]. The increase in fluorescence of SYBR Green was measured after each extension step. C_T_ values were calculated using ABI software. Relative expression values were obtained by normalizing C_T_ values of the tested genes with C_T_ values of the housekeeping genes, using the ΔΔC_T_ method.

### Sequencing

PCR products were prepared for cycle sequencing by gel extraction and purification with the QIAquick Gel Extraction Kit (QIAGEN, Valencia, CA). Cycle sequencing of the purified DNA was carried out with the ABI PRISM Big Dye Terminator v3.1 Cycle Sequencing Kit (Applied BioSystems). The sequencing reactions were processed on an ABI PRISM 3130xl Genetic Analyzer (Applied BioSystems). The sequence analysis was done with the DNA Sequencing Analysis Software, Version 5.2 (Applied Biosystems) for Windows XP and Vista platforms. Sequence alignments were done with the SeqMan II sequence analysis software, version 5.06 (DNASTAR, Inc., Madison, WI).

### Western blot analysis

Cells were removed from cell culture dishes in 15 mM HEPES buffer, pH 7.5, containing 145 mM NaCl, 0.1 mM MgCl_2_, 10 mM ethylene glycol bis (2- aminoethylether) N, N, N, N1–tetraacetic acid (EGTA), 10 μg/ml leupeptin, 10 μg/ml aprotinin, 1 mM phenylmethylsulfonylfluoride (PMSF), and 2 mM sodium orthovanadate (Na_3_VO_4_). Lysates were prepared by freeze thawing, Dounce homogenization, and sonication twice, each time for 15-s intervals at 100 W power. Cell debris was cleared from the lysates by centrifugation at 200× g for 15 min at 4 °C. Lysate fractions were separated into soluble and particulate fractions by centrifugation at 29,000× g for 30 min at 4 °C. The pellets were resuspended in modified RIPA buffer (50 mM Tris-HCl, pH 7.4, 1% Nonident P-40, 0.25% sodium deoxycholate, 150 mM sodium chloride, 1 mM ethylenediaminetetraacetic acid [EDTA], 1 mM PMSF, 5 μg/ml aprotinin, 5 μg/ml leupeptin, 1 μg/ml pepstatin, 1 mM Na_3_VO_4_, and 1 mM sodium fluoride, all purchased from Sigma Aldrich). Protein concentrations were determined with the BCA Protein Assay Reagent Kit (Pierce/Thermo Scientific, Rockford, IL). Both the supernatant and particulate fractions were stored at -80 °C.

Sodium dodecyl sulfate polyacrylamide gel electrophoresis was performed on NuPAGE NOVEX Bis- Tris–4-12% gels with MES SDS running buffer (50 mM Tris base 2-(N-morpholino)ethanesulfonic acid), 1 mm EDTA, 0.1% sodium dodecyl sulfate pH 7.3; Invitrogen). Proteins were detected on the immunoblots with the SuperSignal Femto West Chemiluminescent Substrate Kit (Pierce), according to the manufacturer’s directions. Development of the blots was done with CL-XPosure Film (Pierce), using Kodak GBX developer and fixer solutions (Pierce/Thermo Scientific). Blots were reprobed after removing the antibodies and substrates with the Restore Western Blot Stripping Buffer (Pierce), according to the manufacturer’s directions.

Antibodies used in these studies included: gp91 phox (611415) and p67 phox (69820; BD Biosciences); NOX5 (SC-34707), NOX4 (SC-21860), MOX1/NOX1 (SC-5821), p22 phox (SC-117212), p47 phox (SC-14015 and SC17845), p67 phox (SC-7663), Rac1 (SC-217), and Rac2 (SC-96; Santa Cruz Biotechnology, Santa Cruz, CA); p47 phox (05-540), p40 phox (05-039), and Rac (05-389; Millipore, Bellerica, MA) .

### Assay of NADPH oxidase by superoxide dismutase-inhibitable cytochrome *c* reduction

NADPH oxidase activity was measured in a cell-free system in a manner similar to that used by others [27]. Cells were grown to confluence, rinsed in Delbeccos PBS without Ca^+^ or Mg^++^ (Gibco/Invitrogen, Carlsbad, CA), and harvested with trypsin and EDTA treatment. Trypsin was inhibited with Type I-S soybean trypsin inhibitor (Sigma Aldrich), and the harvested cells were washed with PBS at 4 °C and resuspended in buffer containing 20 mM 3-morpholino propane-1-sulfonic acid and potassium hydroxide (MOPS–KOH buffer, pH 7.4), containing 250 mM sucrose, 0.1 mM EDTA, 2 μM leupeptin, 1 μM aprotinin, and 2 μM pepstatin. The cells were disrupted after one freeze-thaw cycle by two 20-s cycles of Dounce homogenization and two 15-s cycles of sonication at 100 W. Whole cells were removed by centrifugation at 200× g for 5 min at 4 °C. The lysate was fractionated by centrifugation at 29,000g for 15 min at 4 °C. The pellet was then resuspended in 50 mM phosphate buffer at pH 7.4 and stored at -80 °C. Paired assays were conducted by incubation of 20-60 μg of protein with 10 mM phosphate buffer, 130 mM NaCl, 1 mM EGTA, 10 μM flavin adenine dinucleotide (FAD), 2 mM NaN3, 50 μM oxidized cytochrome *c*, and 10 μM guanosine 5' [γ-thio] triphosphate (GTPγS). One reaction of each pair contained 200 units of superoxide dismutase (SOD; Sigma Aldrich). Reactions were initiated by the addition of NADPH to a final concentration of 200 μM. After 1 h, activity was measured as the SOD-inhibitable increase in absorbance at 550 nm (Ε_mM_=21). Assay conditions were established documenting the linearity of superoxide production with time and protein concentrations.

### Superoxide production in whole cells

HCS fibroblasts were grown to the fourth passage in DMEM without phenol red and containing Mito+ and 5% FBS. Cells were harvested using trypsin and EDTA and washed three times with HBSS without Ca and Mg. Cells (10^7^) were resuspended in PBS, pH 7.2, containing 100 μg/ml bovine serum albumin and protease inhibitor cocktail (EDTA free complete protease mix; Roche Diagnostics, Indianapolis, IN). Reduced streptolysin O (5 units/ml; Sigma Aldrich) was added, and the mixture was incubated for 10 min at 37 ^°^C [28]. The permeabilized cells were chilled to 4 ^°^C, harvested by centrifugation, and washed with phosphate buffer, pH 7.2. To measure superoxide production permeabilized cells (1×10^5^ to 1×10^6^) were resuspended in duplicate tubes, with and without 200 units of SOD, containing 30 mM MOPS (pH 7.2), 100 μM FAD, and 100 μM oxidized cytochrome *c*, incubated at room temperature for 5 min, and NADPH added to a final concentration of 200 μM. The change in optical density at 550 nm was recorded every 5 min for 1 h. At the conclusion of the experiment, cells were lysed in 20 mM HEPES buffer containing 1% Triton X-100, and the protein quantitated using the BCA Protein Assay Reagent Kit. Superoxide production was expressed as SOD-inhibitable cytochrome *c* reduction/min/mg protein.

### Small interfering RNA treatment of cells

*NOX1*, *NOX4*, and *NOX5* ON-Target Plus siRNA pools were purchased from Dharmacon (Lafayette, CO). SiRNA complexes were formed in serum-free medium, using INTERFER_IN_ (PolyPlus; Genesee Scientific, San Diego, CA), according to the manufacturer’s instructions. Optimal siRNA/INTERFER_IN_ concentrations were established. Cells were plated at 50-60% confluence in DMEM containing 5% FBS, Mito+serum extender (Gibco, Carlsbad, CA), and ciprofloxacin and maintained at 34 °C. Twelve to 16 h post plating, the media was removed and the cells rinsed with HBSS. The cells were treated with a final concentration of 40 nM siRNA. Cells were transfected with complexes in media composed of INTERFERIN in DMEM containing 5% FBS Mito+ and ciprofloxacin ( in a 1 to 50 ratio) for 16 h. The media was removed and replaced with DMEM with 5% FBS and ciprofloxacin. After 96 h post transfection, the media was removed, the cells were rinsed in PBS, and the RNA was extracted with Trizol, according to the manufacturer’s instructions. The amount of mRNA was determined by real-time PCR, as described above.

### Statistical analysis

Means were compared by one-way analysis of variance, and the significance of differences among means of treatment groups was determined by the Holm-Sidak method (Sigma Stat 3.0; SPSS Inc., Chicago, IL).

## Results

### NADPH-dependent superoxide production by whole cells and membranes

**Figure 1:**
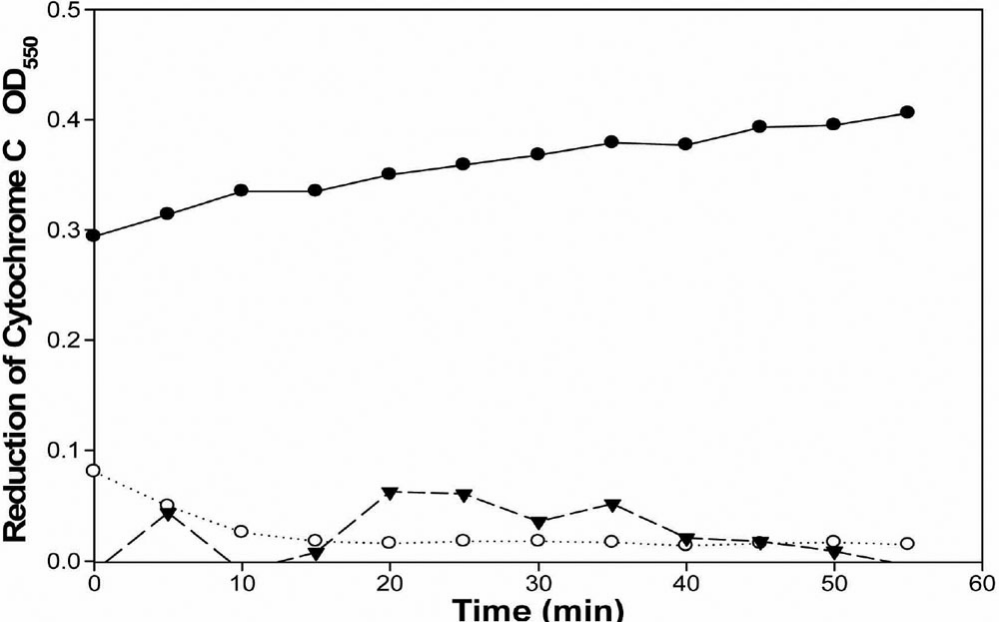
Nicotinamide adenine dinucleotide phosphate-dependent superoxide production by human corneal stromal fibroblasts. Cells were grown in cell culture, harvested, and permeabilized with streptolysin O. The permeabilized cells (2.5×10^5^) were incubated in 3-morpholinopropane-1-sulfonic acid (MOPS) buffer in the presence and absence of 200 units of superoxide dismutase (as described in the Methods). Reactions were initiated by the addition of (●) 200 µM NADPH, (○) 200 µM NADH, or (▼) no substrate. The superoxide dismutase-inhibitable change in optical density at 550 nm (OD_550_) is plotted verses time for 2.5×10^5^ cells.

**Figure 2:**
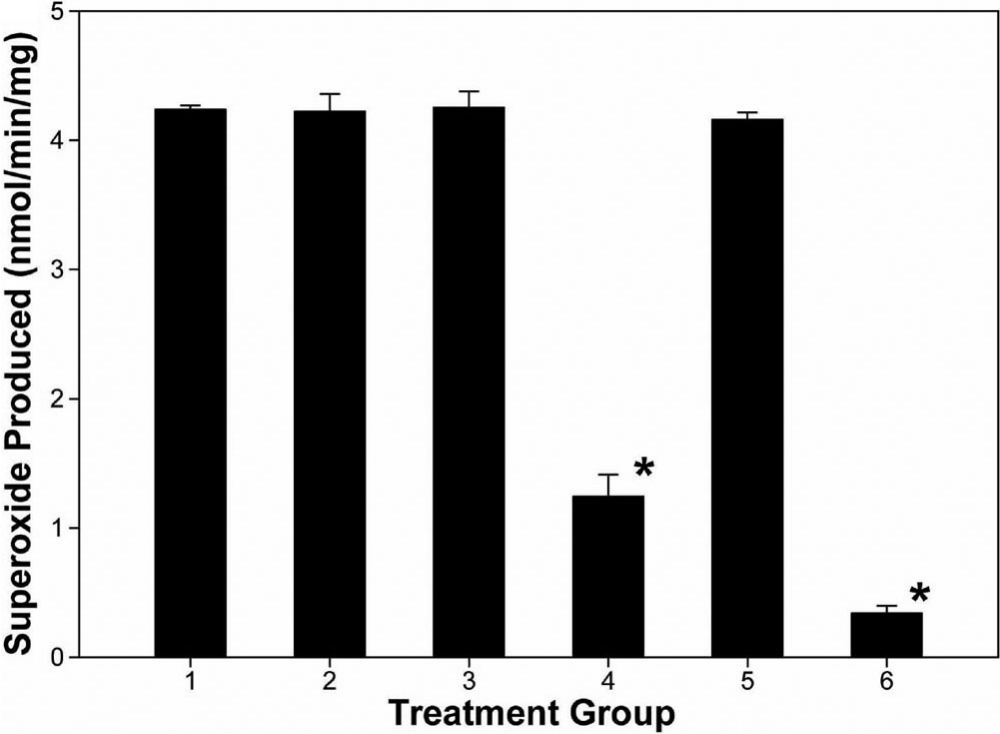
Nicotinamide adenine dinucleotide phosphate oxidase activity in membrane preparations of human corneal stromal fibroblasts as assayed by superoxide dismutase-inhibitable cytochrome c. Human corneal stromal fibroblasts were grown in cell culture, harvested, lysed, and the membranous fraction harvested as the 29,000× g pellet, as described in the Methods. Cell lysates were assayed for superoxide production under various conditions: (1) NADPH as substrate, (2) NADPH as substrate in the presence of 100 μM L-NAME, (3) NADPH as substrate in the presence of 50 µM 1400W, (4) NADPH as substrate in the presence of 10 µM diphenyliodonium, (5) NADPH as substrate in the presence of 100 µM allopurinol, and (6) NADH as substrate. Each bar represents the mean specific activity +/- standard deviation (n=3). An asterisk indicates a significant reduction in SOD inhibitable superoxide production, (p<0.001).

Cultures of HCS cells were grown in serum containing medium as fibroblasts. Cells were harvested, permeabilized, and O_2_^.-^ production was measured as SOD-inhibitable cytochrome *c* reduction. Whole cells permeabilized with streptolysin O produced O_2_^-^ in an NADPH-dependent manner (Figure 1). Membranes prepared from human stromal fibroblasts produced O_2_^.-^ at a rate of 4.24±0.03 nmole/min/mg protein (Figure 2). The addition of inhibitors of nitric oxide synthase, L-NAME or 1400W, or an inhibitor of xanthine oxidase, allopurinol, did not significantly reduce the production of O_2_^.-^, thus indicating that NADPH oxidase was the source of the O_2_^.-^ . NADH did not serve as an effective substrate so the source of the activity was likely not the electron transport chain (Figure 2). The oxidase inhibitor, diphenyliodonium (DPI), reduced activity by 90%, thus indicating that NADPH oxidase was the likely source of O_2_^.-^ production (Figure 2).

### Detection of transcripts of NADPH oxidase and proteins

**Figure 3:**
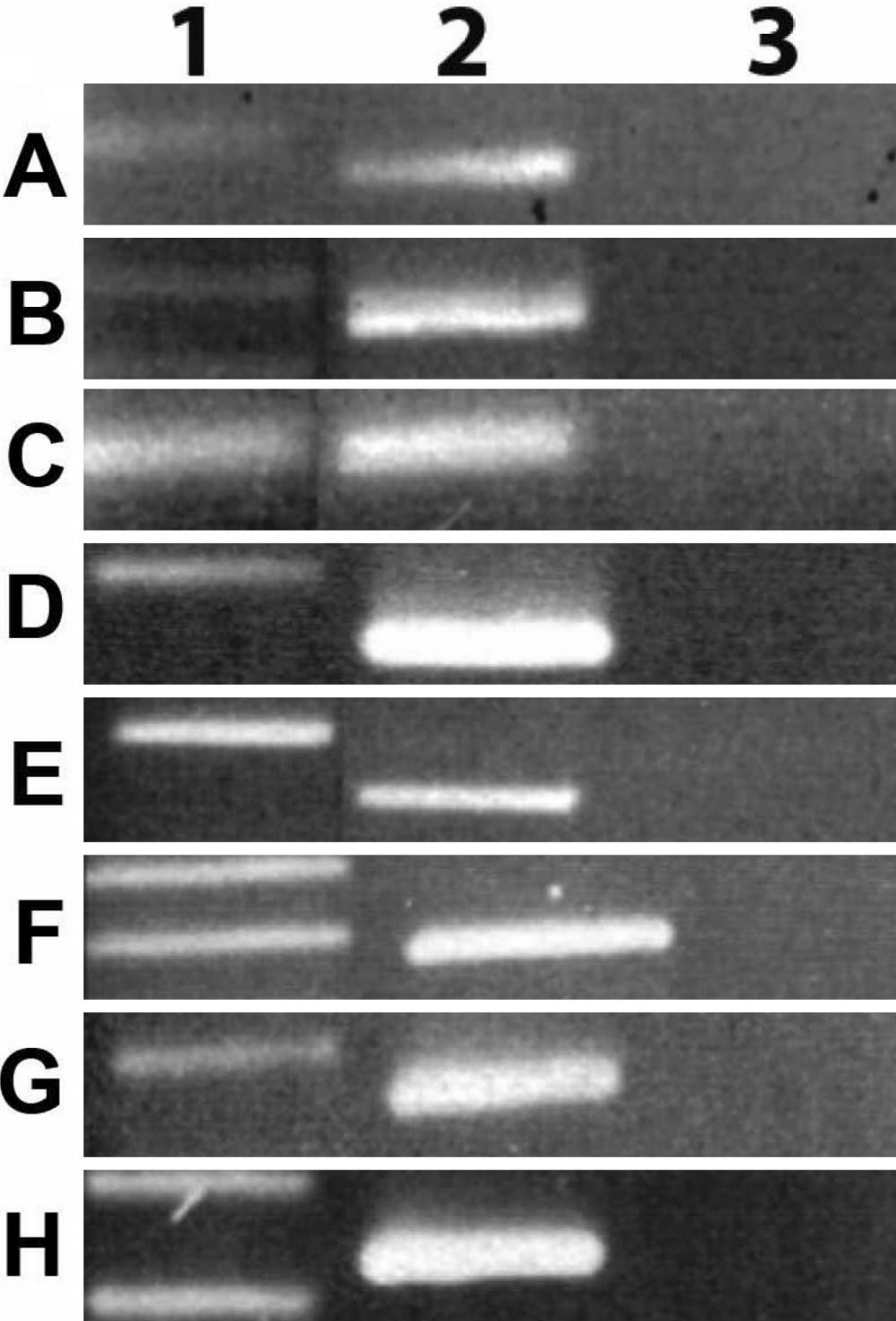
Reverse transcriptase PCR products of NADPH oxidase related genes. HCS fibroblasts were grown to confluence and the RNA extracted from cultures and amplified by RT PCR. Sequence specific primers produced the appropriate PCR products for the NADPH oxidase gene products and Rac including **A**: *NOX1*, **B**: *NOX4*, **C**: *NOX5*, **D**: *p22phox*, **E**: *p47phox*, **F**: *p40phox*, **G**: *p67phox*, and *H*: *Rac*. Row 1: molecular weight standards. Row 2: reverse transcriptase. (RT) PCR products from human corneal stromal fibroblasts; Row 3, no RT controls.

**Figure 4:**
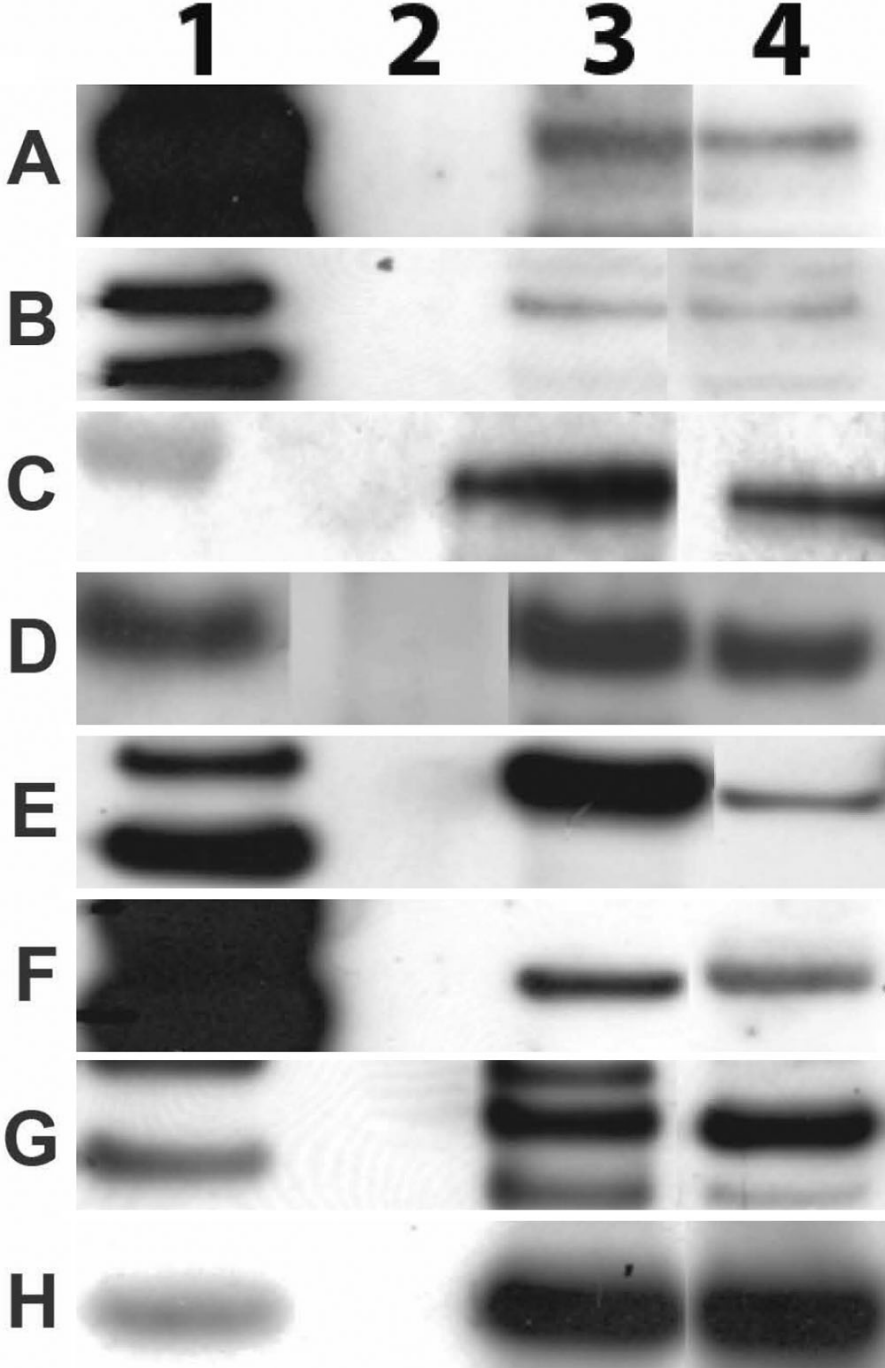
Western blots of proteins from human corneal stromal fibroblasts.  HCS fibroblasts were grown to confluence in DMEM containing 5% FBS. The cell membranes were prepared and lysed in RIPA. The aliquots of proteins were then analyzed by western blotting. The primary antibodies detected proteins of the correct molecular weight; Row 1, Magic Mark molecular weight standards; Row 2, empty; Row 3, positive control protein as detected protein as detected in HL60, HEK or HUVEC cells; Row 4, proteins as detected in HCS fibroblasts. Primary antibodies include **A**: NOX1, **B**: NOX4, **C**: NOX5, **D**: p22 phox, **E**: p47 phox, **F**: p40 phox, **G**: p67 phox, and **H**: Rac.

Cultures of human stromal fibroblasts expressed three *NOX* transcripts when grown in DMEM containing 5% FBS. PCR reactions containing cDNAs prepared from cultures of stromal cells produced the appropriate products from primers for *NOX1*, *NOX4*, and *NOX5* (Figure 3A-C). RT PCR using *NOX4* primers amplified a region of the *NOX4* coding sequence, including bp 1686 to 1855, which was 98.0% identical to that originally discovered in cells from the kidney, NM_016931.2 [14]. The *NOX5* RT PCR product was 99.0% identical to a region encoding a portion of the EF hand /Ca binding region of the *NOX5* from human testes NM_024505 [29]. Western blots using antibodies to NOX1, NOX4, and NOX5 confirmed that these NOX proteins were expressed in HCS fibroblasts (Figure 4A-C).

Cultured HCS fibroblasts also produced transcripts of other proteins required to form functional NADPH complexes with NOX1 and NOX4 (Figure 3). HCS fibroblasts did express mRNAs for *p22 phox*, *p47 phox*, *p40 phox*, and *p67 phox,* as detected by RT-PCR reactions using sequence-specific primers (Figure 3D-H). HCS fibroblasts also constitutively expressed Rac1, as amplified with *Rac1*-specific primers (Figure 3H).

Western blots using antibodies to p22 phox did detect the production of a protein of the correct molecular weight for p22 phox (Figure 4D). A single dominant band of protein at about 47 kDa was detected with antibodies to p47 phox (Figure 4E). Western blots prepared with antibody to p40 phox and proteins extracted from HCS fibroblasts showed a band at 44 kDa when probed with the monoclonal antibody clone 1.9 (Millepore; Figure 4F). Blots prepared using mouse monoclonal antibody to p67 phox detected a dominant band of protein at 65-67 kDa, indicating that p67 phox was constitutively produced (Figure 4G). Western blots using antibodies to Rac produced a 21 kDa band in membrane containing fractions of HCS fibroblasts (Figure 4H). While these data do not prove that Rac is bound to the complex of NADPH oxidase proteins in the membrane, the data indicate that NOX1 and other oxidases that require Rac have the potential to be active.

### Small interfering RNA suppression of NADPH oxidase activity

**Figure 5:**
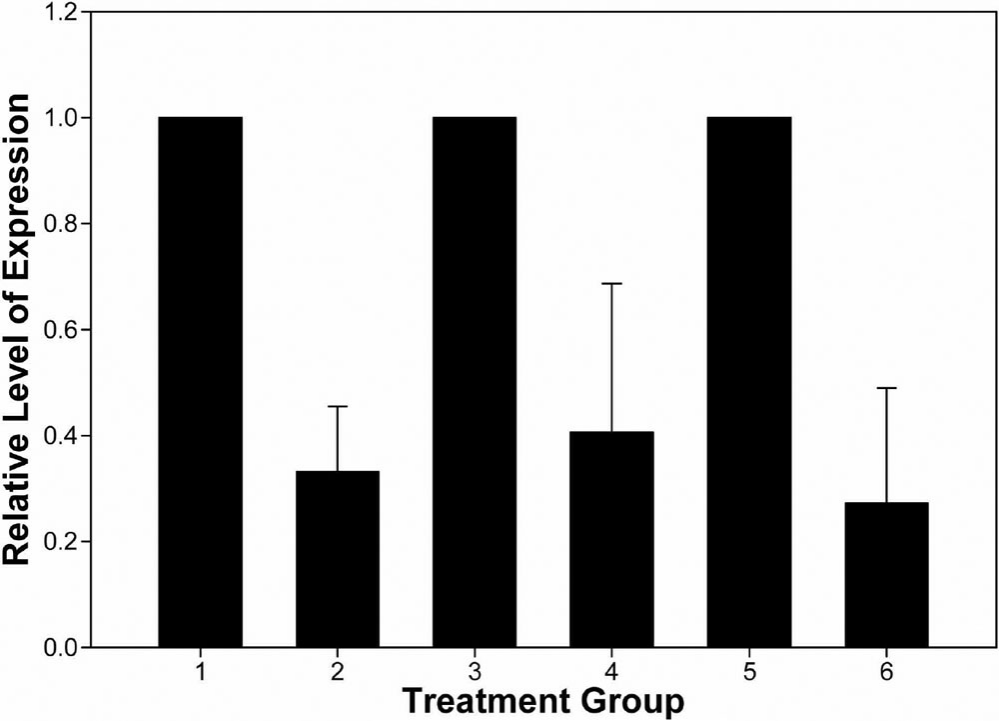
Relative mRNA content of cells treated with small interfering RNAs to *NOX1*, *NOX4*, or *NOX5*. Human corneal stromal  fibroblasts were treated with 40 nM small intefering (si)RNAs to nontarget controls (lanes 1,3, and 5) or *NOX1*-specific (lane 2), *NOX4*-specific (lane 4), or *NOX5*-specific (lane 6) siRNAs. RNA was extracted and amplified in triplicate amplification by real-time reverse transcriptase PCR, using the SYBR Green. Each bar represents the mean and standard error of the mean of triplicate amplifications of RNAs extracted from triplicate cultures. A significant reduction (p<0.05) in the steady state level of RNA was observed for each specific mRNA relative to cultures treated with non-target control RNAs.

HCS fibroblasts were treated with siRNAs to *NOX1*, *NOX4*, or *NOX5* to confirm the existence of these multiple forms of NOX. HCS fibroblasts were treated with siRNA smart pools or nontarget control siRNAs, as described in the Methods section. Real-time PCR documented reductions in the steady state mRNA pools by 67% for *NOX1*, 59% for *NOX4*, and 73% for *NOX5* at 96 h post treatment (Figure 5).

## Discussion

HCS fibroblasts produced O_2_^.-^, as detected by NADPH-dependent, SOD-inhibitable, cytochrome *c* reduction. Membranes prepared from these cells retained the ability to produce O_2_^.-^ in a manner that was inhibitable by DPI but that was insensitive to L-NAME and allopurinol. NADPH was the preferred substrate, while NADH was a poor substrate. These observations indicate that the source of the O_2_^.-^ was not NOS, xanthine oxidase, or the mitochondrial electron transport chain. The rate of O_2_^.-^ production, the dependence upon NADPH as the substrate, and the specific activity of O_2_^.-^ production were indicative that NADPH oxidase was the source of O_2_^.-^.

HCS fibroblasts expressed three NOX genes as well as five genes whose products serve as accessory proteins that compose O_2_^.-^-producing NADPH complexes. RT-PCR products generated with gene-specific primers and the proteins detected by western blots documented the expression of NADPH oxidase components. SiRNA knockdowns of mRNA pools confirm the expression of the NOX mRNAs. *NOX1*, *NOX4*, and *NOX5* mRNAs were produced in a constitutive manner at detectable levels when cells were grown in serum containing medium. *NOX2* mRNA was detected, but no protein was found despite the use of multiple NOX2 antibodies along with positive and negative controls. In addition, neither *NOX3* mRNA nor protein was detected. The literature now indicates that it is not unusual for a given cell type to express multiple NOXs [4,30]. It has been hypothesized that each isoform of NOX may function in a specific manner by virtue of its localized production of O_2_^.-^ [1]. Future studies will need to document the subcellular localization and function of these NADPH oxidases in corneal stromal cells.

Among the three isoforms of NOX present in HCS fibroblasts, NOX5 produces O_2_^.-^ in a strictly NADPH-dependent manner when bound to the cytoplasmic membrane [31,32]. The accessory proteins are not required for activity of NOX5 [33]. NOX5 has been reported to exist in any of five isoforms (α, β, γ, δ, and ε) [34]. The literature indicates that the molecular weight of NOX5 may range from 65 to 85 kDa, depending on the isoform present and the extent of glycosylation [31]. The *NOX5* gene encodes a protein with a molecular weight of 64.7 kDa, which is close to the estimated molecular weight of the protein that we detect with the NOX5 antibody [14]. The fact that NOX5 siRNA reduced the production of mRNA indicates that *NOX5* is actively expressed in HCS fibroblasts.

Unlike NOX5, NOX4 requires p22 phox for stabilization and activity [5]. NOX4 does not require other accessory proteins for activity [4]. NOX4 appears to localize in membranes of cells, such as the cytoplasmic membrane and endoplasmic reticulum, but its localization appears cell-type dependent [35]. The data suggest that in some cells the activity of NOX4 is Rac dependent [36]. NOX4 can be active when complexed with p22 phox alone, but other studies indicate that accessory proteins present in the cytoplasm can be activated and complex with NOX4 and p22 phox to regulate NOX4 activity [33]. A variety of agents have been documented to activate NOX4, including lipopolysaccharides, cytokines, growth factors, angiotensin II, phorbol mynistic acid (PMA), and insulin [4]. Each activating stimuli has been observed to have its effects in a cell-type and sometimes species-dependent fashion. NOX4 has a predicted molecular weight of 66.9 kDa and is often detected as a protein of about 65 kDa by western blots, as we have detected [14]. NOX4-targeted siRNA treatment of HCS fibroblasts reduced the pools of mRNA, confirming the existence of the *NOX4* message.

The NOX1 homologue has been detected in a wide variety of cell types and tissues. NOX1 has been observed in three isoforms, but the full-length gene encodes a protein in the range of 55-60 kDa, as we have detected in HCS fibroblasts [4]. The subcellular localization of NOX1 is not well defined as it seems to vary in a cell-type-dependent manner. O_2_^.-^ produced as a result of NOX1 activity has been observed to enhance proliferation of some cell types while inhibiting the growth of other cells [37,38]. Unlike NOX3 and 5, NOX1 gene expression and activity is regulated by growth factors and cytokines [4]. *NOX1* mRNA was detected, and a NOX1 antibody-reactive protein of about 65 kDa was detected in membrane fractions of HCS fibroblasts. NOX1 requires p22 phox and an organizer protein, like NOXO1 or p47 phox, as well as a NOX activator protein like NOXA1 or p67 phox, and Rac for efficient O_2_^.-^ production [39]. Treatment of cells with *NOX1* siRNA suppressed *NOX1* mRNA accumulation, documenting the existence of *NOX1* mRNA pools within HCS fibroblasts. It remains to be determined how NOX1 activity is regulated in HCS fibroblasts and what the biological activity is of the O_2_^.-^ it produces.

HCS fibroblasts also expressed mRNAs encoding p22 phox, p47 phox, p67 phox, and Rac. These proteins are either required or regulate the activities of NOX1, 2, 3, and 4 oxidases [13,33]. Each of these proteins has specific functions. P22 phox interacts with the NOX proteins and becomes part of a membrane-bound electron transfer complex know as cytochrome *b*_558_ [13]. HSC fibroblast constitutively expressed *p22 phox* mRNA and western blots with two different antibodies, indicate that the protein was present in the cytosol and membrane fractions. A second accessory protein required for NOX2 and NOX1 activity is p47 phox, the organizer protein [33]. P47 phox binds to the p22 phox/ NOX complex via the Src homology domain and to membranes by a PX domain [40,41]. It has been reported that in some cells p47 phox can be replaced functionally by a p47 phox homologue, NOXO1 (NOX organizer1) [42]. Based on the primers that we used and the antibodies available to us, it is not clear that we could distinguish between p47phox and its homologue. It appears that HCS fibroblasts produce p47 phox. A third protein that is known to activate the classic NOX2 complex and may modify the activity of NOX1, NOX3, and NOX4 complexes is p67 phox. A p67 phox homologue, NOXA1 (NOX activator) contributes to the activation of NOX1 in some cells [40]. P67 phox is expressed in HCS fibroblasts. A fourth protein believed to contribute to the activity of some NADPH oxidases is p40 phox. P40 clearly influences the activity of NOX2 NADPH oxidases, but it has not been clearly established to what extent it affects the activity of NOXs 1 and 3 [4]. P40 phox binds to p47 phox and p67 phox in NOX1 and NOX2 NADPH oxidase complexes. Our data clearly demonstrated its expression in HCS fibroblasts. While p40 phox is contained within membranous fractions, it does not appear to be required for activity of NOX1, based on the work of others [4]. Rac, another protein that appears to be required for full activity of NOX1, 2, and 3 and which may influence the activity of NOX4 oxidases was produced and was bound to membrane fractions in HCS fibroblasts [39,43,44].

HCS fibroblasts produce O_2_^.-^ in a constitutive manner consistent with NADPH oxidase activity. mRNAs and proteins required to form functional NOX1, NOX4, and NOX5 NADPH oxidases were detected in these cells. Membranous fractions prepared from HCS fibroblasts produced O_2_^.-^ in an NADPH-dependent manner that was consistent with NADPH oxidase activity. Both RT PCR and western blotting indicate that the accessory proteins p22 phox , p47 phox, p67 phox, p40 phox, and and Rac are all expressed in HCS fibroblasts. Treatment of cells with siRNAs for *NOX1*, *NOX4*, and *NOX5* reduced the amount of each respective mRNA in cells. NOX and accessory proteins were present in membranous fractions prepared from cells, as we have described. The functions of the O_2_^.-^ produced by each of the NOX oxidases remain to be discovered.
